# RNA-seq reveals downregulated osteochondral genes potentially related to tibia bacterial chondronecrosis with osteomyelitis in broilers

**DOI:** 10.1186/s12863-020-00862-2

**Published:** 2020-06-03

**Authors:** Haniel Cedraz de Oliveira, Adriana Mércia Guaratini Ibelli, Simone Eliza Facioni Guimarães, Mauricio Egídio Cantão, Jane de Oliveira Peixoto, Luiz Lehmann Coutinho, Mônica Corrêa Ledur

**Affiliations:** 1 Universidade Federal de Viçosa, Viçosa, Minas Gerais Brazil; 2 Embrapa Suínos e Aves, Rodovia BR-153, Km 110, Distrito de Tamanduá, Caixa Postal: 321, Concórdia, Santa Catarina 89715-899 Brazil; 3 Programa de Pós-Graduação em Ciências Veterinárias, Universidade Estadual do Centro-Oeste, Guarapuava, Paraná Brazil; 4 Universidade de São Paulo, Piracicaba, SP Brazil; 5 Programa de Pós-Graduação em Zootecnia, UDESC-Oeste, Chapecó, SC Brazil

**Keywords:** RNA-Seq, *Gallus gallus*, BCO, Lameness, Cartilage

## Abstract

**Background:**

Bacterial chondronecrosis with osteomyelitis (BCO) develops in the growth plate (GP) of the proximal femur and tibia and is initiated by damage to the less mineralized chondrocytes followed by colonization of opportunistic bacteria. This condition affects approximately 1% of all birds housed, being considered one of the major causes of lameness in fast growing broilers. Although several studies have been previously performed aiming to understand its pathogenesis, the molecular mechanisms involved with BCO remains to be elucidated. Therefore, this study aimed to generate a profile of global differential gene expression involved with BCO in the tibia of commercial broilers, through RNA sequencing analysis to identity genes and molecular pathways involved with BCO in chickens.

**Results:**

Our data showed 192 differentially expressed (DE) genes: 63 upregulated and 129 downregulated in the GP of the tibia proximal epiphysis of BCO-affected broilers. Using all DE genes, six Biological Processes (BP) were associated with bone development (connective tissue development, cartilage development, skeletal system development, organ morphogenesis, system development and skeletal system morphogenesis). The analyses of the upregulated genes did not indicate any significant BP (FDR < 0.05). However, with the downregulated genes, the same BP were identified when using all DE genes in the analysis, with a total of 26 coding genes explaining BCO in the tibia: *ACAN, ALDH1A2, CDH7, CHAD, CHADL, COL11A1, COMP, CSGALNACT1, CYR61, FRZB, GAL3ST1, HAPLN1, IHH, KIF26B, LECT1, LPPR1, PDE6B, RBP4A, SERINC5, SFRP1, SOX8, SOX9, TENM2, THBS1, UCHL1* and *WFIKKN2.* In addition, seven transcription factors were also associated to BCO: *NFATC2, MAFB, HIF1A-ARNT, EWSR1-FLI1, NFIC, TCF3* and *NF-KAPPAB*.

**Conclusions:**

Our data show that osteochondral downregulated genes are potential molecular causes of BCO in broilers, and the bacterial process seems to be, in fact, a secondary condition. Sixteen genes responsible for bone and cartilage formation were downregulated in BCO-affected broilers being strong candidate genes to trigger this disorder.

## Background

The broiler chickens have undergone intense genetic and nutritional improvement, exponentially increasing their weight gain and decreasing the slaughter age. However, the improvement focused on the high performance has overlooked some physiological characteristics, such as the skeletal structure [1]. The bacterial chondronecrosis with osteomyelitis (BCO) affects up to 1% of all birds housed, being considered a worldwide major cause of lameness in commercial broilers, generating economic losses and impacting negatively the animal welfare [2–4]. The prevalence of lameness due the BCO can extent up to 50%, and 5% of mortality [5], however the incidence of BCO is unknown in most countries [2–4, 6].

BCO affect the chicken GP, where the chondrocyte columns are irregularly aligned due to the high longitudinal growth rates, being associated with the growth plate turnover in this specie [7]. The BCO pathogenesis is supposed to be initiated by the damage of the poorly mineralized chondrocytes followed by colonization of opportunistic bacteria, such as *Staphylococcus aureus*, *Escherichia coli*, *Coagulase-negative Staphylococcus* and *Enterococcus* spp., in the osteochondrotic clefts [2, 6, 7] of both femur and tibia [8, 9].

Despite the fact that many opportunistic microorganisms have been identified in studies focused on the involvement of bacteria in the process of BCO [3, 6–12], there are no studies exploring the genetics and molecular mechanisms involved with BCO in tibia, although, previous studies have shown the importance of some genes involved with BCO in the femur [13–15]. Therefore, the aim of this study was to identify the global differential gene expression profile in the tibia GP involved with BCO in chickens, through the transcriptome analysis using RNA-seq of normal and BCO-affected broilers.

## Results

### RNA-Seq data

An average of 17,5 million reads/sample (2 × 100 base paired-end reads) was generated and about an average of 13 million reads/sample were kept after the data quality control. The transcriptome had in average more than 99% of the reads mapped against the chicken reference genome Galgal5, ranging from 98.83 to 99.26% to each individual sample, with 73% of the reads present in genes.

### Differential gene expression

A total of 12,576 transcripts were obtained. From those, 1018 transcripts were selected with FDR < 0.05, and grouped in three categories: protein-coding (973), lncRNA (44) and snoRNA (1). The transcripts with logFC < − 2.0 and > 2.0 were selected, obtaining 192 DE genes (12 lncRNA and 180 protein-coding), being 63 upregulated (logFC> 2.0) (8 lncRNA and 55 protein-coding) and 129 downregulated (logFC<− 2.0) (4 lncRNA and 125 protein-coding (112 annotated genes) in the affected compared to healthy broilers (Additional File 1).

### Gene ontology

In the first step, 192 DE genes were used in the GO analysis and a total of 304 Biological Processes (BP) were identified. From those, only nine BP were significant with FDR ≤ 0.05 (Table 1). Among these nine BP, only six were specifically related to bone development: connective tissue development, cartilage development, skeletal system development, organ morphogenesis, system development, and skeletal system morphogenesis.

**Table 1:** Biological Process (BP) found using DAVID 6.8. Table 1a shows nine BP using all 192 Differentially Expressed (DE genes) with six BP associated with bone formation. Table 1b shows six BP using only 129 downregulated genes in the affected broilers. In bold are the BP that were the same in both analyses

Category	Term	Pathways	Count	FDR
Table 1a**.**				
**GOTERM_BP_ALL**	**GO:0061448**	**connective tissue development**	**12**	**1.89E-05**
**GOTERM_BP_ALL**	**GO:0051216**	**cartilage development**	**11**	**2.46E-05**
**GOTERM_BP_ALL**	**GO:0001501**	**skeletal system development**	**15**	**3.81E-05**
**GOTERM_BP_ALL**	**GO:0009887**	**organ morphogenesis**	**17**	**6.34E-04**
**GOTERM_BP_ALL**	**GO:0048731**	**system development**	**35**	**0.00825838**
**GOTERM_BP_ALL**	**GO:0048705**	**skeletal system morphogenesis**	**9**	**0.008659783**
GOTERM_BP_ALL	GO:0007275	multicellular organism development	36	0.019958649
GOTERM_BP_ALL	GO:0048856	anatomical structure development	39	0.026663753
GOTERM_BP_ALL	GO:0044707	single-multicellular organism process	40	0.031747676
Table 1b**.**				
**GOTERM_BP_ALL**	**GO:0001501**	**skeletal system development**	**15**	**2.31567E-07**
**GOTERM_BP_ALL**	**GO:0061448**	**connective tissue development**	**12**	**3.26095E-07**
**GOTERM_BP_ALL**	**GO:0051216**	**cartilage development**	**11**	**6.13948E-07**
**GOTERM_BP_ALL**	**GO:0048705**	**skeletal system morphogenesis**	**9**	**0.000509513**
**GOTERM_BP_ALL**	**GO:0009887**	**organ morphogenesis**	**14**	**0.001261476**
**GOTERM_BP_ALL**	**GO:0048731**	**system development**	**26**	**0.049749525**

In a second step, the GO analyses were performed separating the up and downregulated genes. Using the 63 upregulated genes, the GO did not show any significant BP. However, when the 129 downregulated genes were used, the GO showed the same six BP that were identified when all 192 DE genes were used, with a total of 26 coding genes among the BPs (Table 2). The most enriched BP were system development (26 genes), skeletal system development (15 genes), organ morphogenesis (14 genes), connective tissue development (12 genes), cartilage development (11 genes) and skeletal system morphogenesis (9 genes) (Table 1).

**Table 2:** Twenty-six downregulated genes associated with bone formation enriched in the Biological Processes found using DAVID 6.8 tool

Gene symbol	Gene ID	logFC	FDR	Gene Name
*TENM2*	ENSGALG00000001768	−4.61	4.17E-05	teneurin transmembrane protein 2
*WFIKKN2*	ENSGALG00000007367	−4.32	1.66E-06	WAP, follistatin/kazal, immunoglobulin, kunitz and netrin domain containing 2
*RBP4A*	ENSGALG00000006629	−3.83	4.45E-09	retinol binding protein 4
*LECT1*	ENSGALG00000016945	−3.19	3.99E-03	chondromodulin
*COMP*	ENSGALG00000003283	−3.18	4.80E-02	cartilage oligomeric matrix protein
*UCHL1*	ENSGALG00000014261	−3.12	1.39E-10	Ubiquitin C-Terminal Hydrolase L1
*ACAN*	ENSGALG00000006725	−3.09	3.72E-03	aggrecan
*CDH7*	ENSGALG00000013782	−3.05	3.04E-05	cadherin 7
*IHH*	ENSGALG00000011347	−3.00	3.10E-04	Indian hedgehog protein
*HAPLN1*	ENSGALG00000015627	− 2.97	1.51E-03	Hyaluronan and proteoglycan link protein 1
*KIF26B*	ENSGALG00000010664	− 2.55	2.62E-02	kinesin 32 Family member 26B
*PLPPR1*	ENSGALG00000015542	−2.52	2.02E-05	phospholipid phosphatase related 1
*COL11A1*	ENSGALG00000005180	−2.47	1.51E-02	Collagen Type XI Alpha 1 Chain
*GAL3ST1*	ENSGALG00000007781	−2.45	1.19E-02	galactose-3-O-sulfotransferase 1
*SOX9*	ENSGALG00000004386	−2.40	4.58E-05	SRY-box 9
*SFRP1*	ENSGALG00000003473	−2.37	1.47E-03	secreted frizzled related protein 1
*CHADL*	ENSGALG00000011970	−2.34	2.48E-07	chondroadherin like
*CSGALNACT1*	ENSGALG00000010125	−2.33	4.59E-06	chondroitin sulfate N-acetylgalactosaminyltransferase 1
*FRZB*	ENSGALG00000002763	−2.33	1.35E-04	frizzled-related protein
*CYR61*	ENSGALG00000008661	−2.28	3.63E-07	Protein CYR61
*SOX8*	ENSGALG00000005263	−2.27	1.29E-04	SRY-box 8
*THBS1*	ENSGALG00000009626	−2.22	2.35E-02	thrombospondin-1 precursor
*CHAD*	ENSGALG00000019761	−2.12	7.28E-03	chondroadherin
*PDE6B*	ENSGALG00000015373	−2.11	1.67E-03	phosphodiesterase 6B
*ALDH1A2*	ENSGALG00000004270	−2.11	1.60E-12	Retinal dehydrogenase 2
*SERINC5*	ENSGALG00000014798	−2.09	6.48E-05	serine incorporator 5

### Gene and Transcription factor (TF) network

**Fig. 1:**
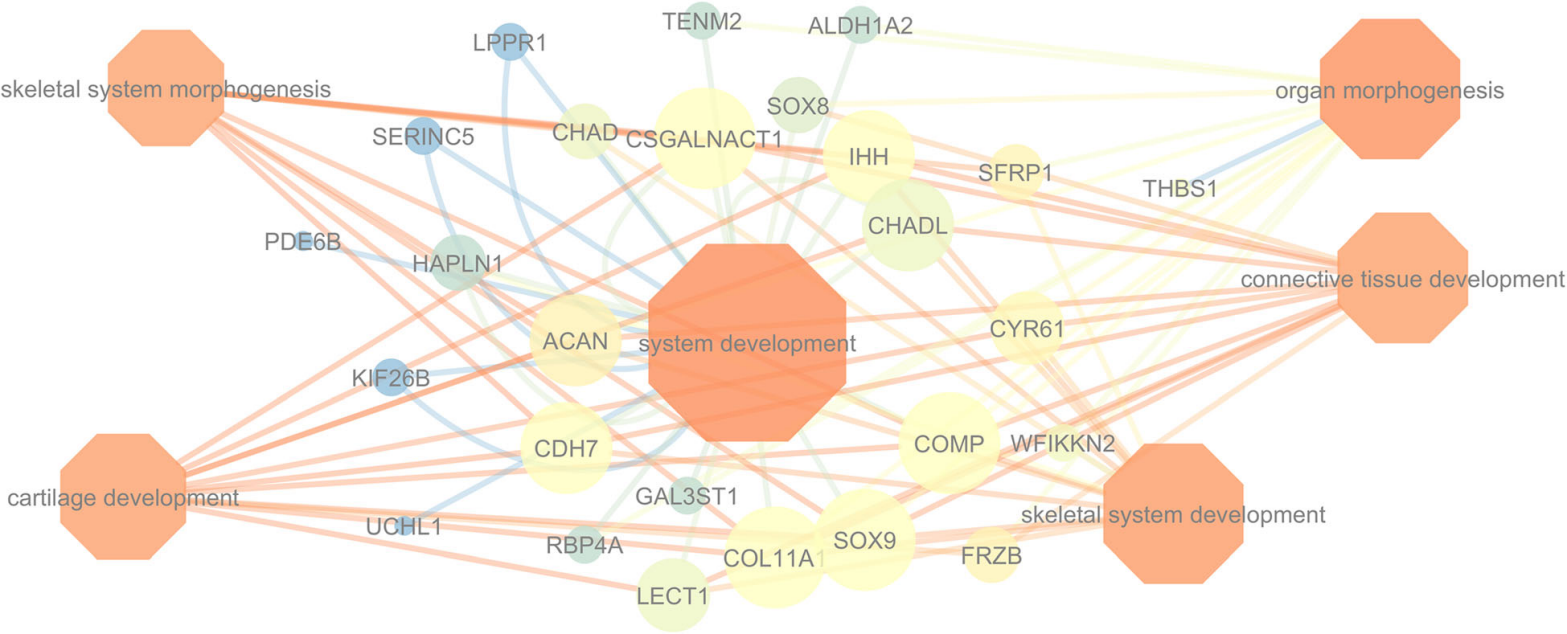
Gene network showing connections between downregulated genes (circles) and Biological Process (diamonds). The size (low values are small size) and the colors (low values are in bright colors) of the nodes indicate the number of directed edges and the neighborhood connectivity, respectively. The Edge colors indicate the betweenness of the edges (low values are in bright colors)

**Fig. 2:**
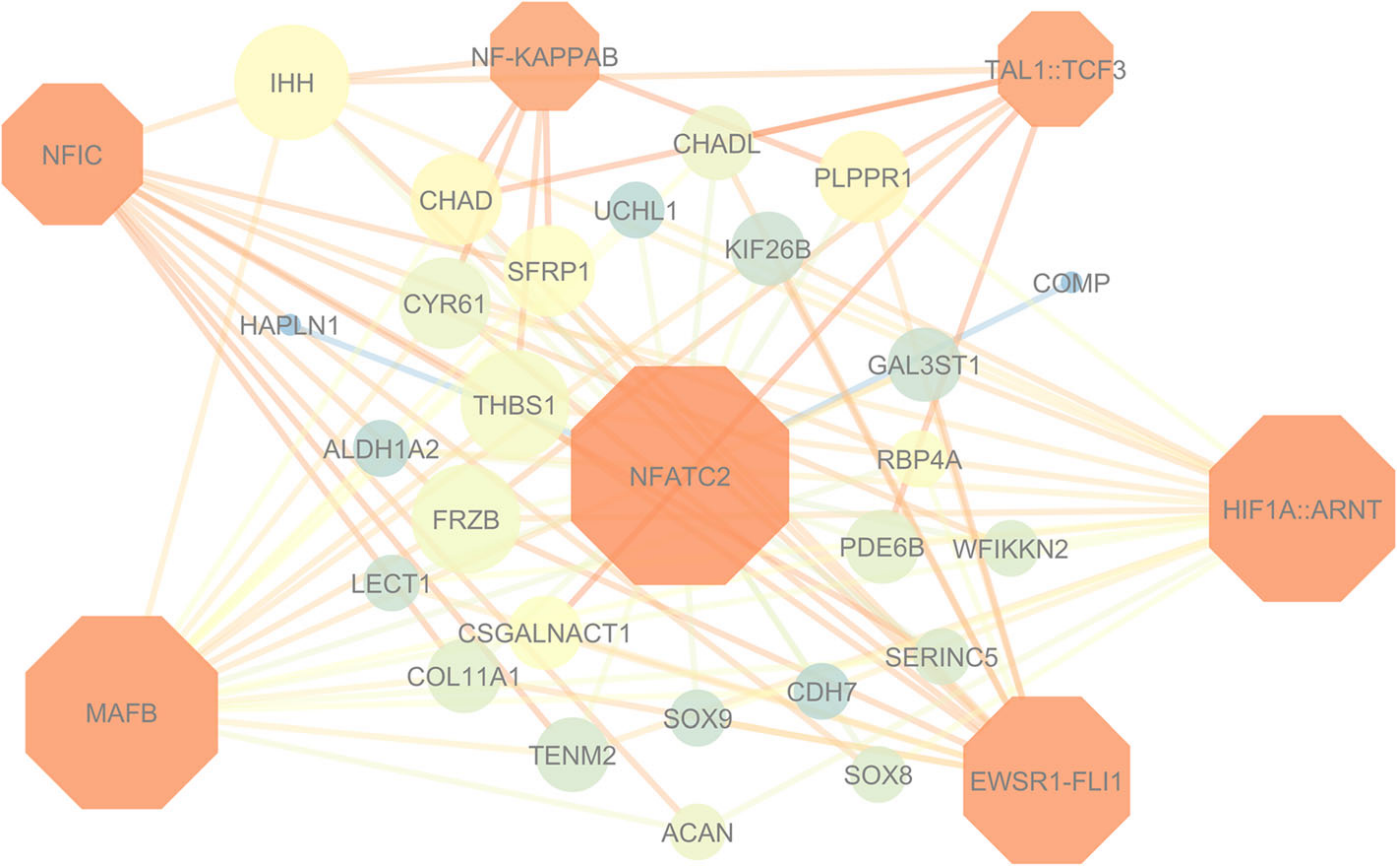
Transcription Factor network showing connections between downregulated genes (circles) and Transcription Factors (diamonds). The size (low values are small size) and the colors (low values are in bright colors) of the nodes indicate the number of directed edges and the neighborhood connectivity, respectively. The Edge colors indicate the betweenness of the edges (low values are in bright colors)

A gene network using Cytoscape v.3.6 [16]. was performed to identify connections among genes enriched in bone bioprocesses (Fig. 1). Three genes (*COMP, COL11A1* and *SOX9*) were shared in all six BP, four genes (*CDH7, CSGALNACT1*, *ACAN* and *IHH*) were shared by five BP, three genes (*CHADL, CYRG1* and *LECT1*) were shared by four BPs, four genes (*SFRP1, CHAB, FRZB* and *SOX8*) were shared by three BPs and three genes (*TENM2, WFIKKN2* and *ALDH1A2*) were shared by two BPs (Additional file 2). The other genes were in only one BP (Fig. 1). In addition, regulatory sequence analyses for all detected genes were performed for the 26 genes used as input at TFM-explorer [17]. A total of 21 transcription factors (TF) were identified from the downregulated genes. Based on the BP from DAVID and the literature review, we selected the most putative BCO associated TF. The main TF, from the most to the least connected were: *NFATC2, MAFB, HIF1A::ARNT, EWSR1-FLI1, NFIC, TCF3 and NF-KAPPAB*, which were used to construct a TF network highlighting the most connected genes (Fig. 2).

## Discussion

Tibia is a high-mineralized bone in the body, being considered a good indicator of mineralization in ossification studies [18, 19]. The bacterial chondronecrosis with osteomyelitis (BCO) has been diagnosed worldwide and its pathogenesis seems to be related to the poor mineralization of chondrocytes and cartilage of the femur and tibia bones in fast-growing chicken, with proliferation of opportunistic bacteria [7]. Although several studies are available evaluating femur, it is known that the lesion status differ between bones [3], and that high incidences of tibia BCO has been previously reported in broilers [20]. Nowadays, there are just few studies addressing the molecular mechanisms involved with femur BCO [13–15] and no information regarding tibia BCO is available which highlights the importance of our study, since it brings novel and relevant knowledge to the field. Furthermore, this condition is observed in males and females, and an influence of sire line on susceptibility for this trait has already been found [20]. However, it is consensus that high performance broilers are widely affected by BCO [3, 20–22], which reinforces the importance of new studies considering tibia BCO.

Although only one commercial line was used in this study, it is expected common molecular pathways involved in the BCO development in different lines, since this is a health condition. However, the line or sex effect could influence the gene expression profile related to this condition, leading to a variation in the BCO manifestation, and should be further confirmed. According to Wideman et al. (2012) [3], no difference was found on the incidence of lameness between males and females and among lines in the same type of floor. Regarding tibia BCO, no differences between males and females were found until 40 days, but an effect of sire was observed, especially when broilers were older than 6 weeks of age [20]. In our study, we used males from a high performance commercial broiler line since they are fast growing, heavy weight and therefore more probable to get affected with BCO. Therefore, if we have used other high performance commercial line, we expect that similar results would be obtained.

In the tibia GP transcriptome, 1018 genes were DE between normal and BCO-affects broilers. A total of 44 lncRNA were identified and these transcripts may be related to the regulation of adjacent genes [23]. According to the position in the genome of the NCBI database, the lncRNAs ENSGALG00000040226, ENSGALG00000033872 and ENSGALG00000032326 are close to the genes *SOX9, COL11A1* and *SOX8*, respectively, and may be involved in the regulation of these important genes for bone development. The *SOX9* and *SOX8*, two transcriptions factors acting on endochondral ossification, have recently been associated to femur epiphysiolysis [15]. In the current study, it was possible to identify several DE genes involved with BCO development, even using a small number of samples per group, as can be seen in several RNA-Seq studies, when groups are well characterized [24–28].

Initially, using 112 downregulated genes, a total of 26 genes enriched six BP directly related to bone and cartilage development (Table 2): system development (GO: 0048731), skeletal system development (GO: 0001501), organ morphogenesis (GO: 0009887), connective tissue development (GO: 0061448), cartilage development (GO: 0051216) and skeletal system morphogenesis (GO: 0048705). These are important BP for bone development and their malfunction can cause poor ossification, leading to cases of lameness, commonly observed in BCO-affected chickens [12]. The genes related to these biological pathways are *ACAN, ALDH1A2, CDH7, CHAD, CHADL, COL11A1, COMP, CSGALNACT1, CYR61, FRZB, GAL3ST1, HAPLN1, IHH, KIF26B, LECT1, LPPR1, PDE6B, RBP4A, SERINC5, SFRP1, SOX8, SOX9, TENM2, THBS1, UCHL1* and *WFIKKN2* (Fig. 1). These are functional candidate genes associated to BCO, as discussed below.

The aggrecan gene (*ACAN*) is one of the most important cartilage aggregating chondroitin [29]. This gene is essential to the regulation of growth factors and cartilage development [30, 31], and it is associated with diseases such as skeletal dysplasia [32], osteoarthritis [33], osteochondritis [34, 35] and chondrodystrophy in chickens [36]. In addition, *SOX8* and *SOX9* play an important role as transcription factors in the development of cartilage, controlling the differentiation of chondrocytes and osteoblasts in the development of progenitor cells [30, 31, 37–42].

The *CHAD* gene (Chondroadherin) is highly expressed in cartilage and was first isolated in cattle [43], direct binding with calcium phosphate [44]. Hessle et al. (2013) [38] showed that *CHAD* has an influence on the enlargement of the epiphyseal plate and its inactivation compromises the hypertrophic differentiation of the chondrocytes, as well as the cartilage composition in rats. An important *CHAD* paralog is *CHADL*, which can negatively regulate chondrocyte differentiation, inhibiting cartilage fibrillogenesis and can be used as a marker for cartilage diseases [45].

Collagen type XI (*COL11*) constitutes a triple helix formed by *COL11a1*, *COL11a2* and *COL12a1*, being an essential component of the extracellular matrix-regulating the diameter of the fibrils [46–49]. In the absence of *COL11a1*, an alternate triple helix is formed with *COL11a2* and *COL5a1*, but it is unable to compensate for the functional deficiency of Collagen 11a1 [50]. *COL11* is essential for the cartilage collagen fibrils formation and for differentiation and organization of growth plate chondrocytes [51]. The *COL11a1* plays a key role in endochondral ossification, and the low expression of this gene results in alterations in the mineralization of newly formed bone [40].

The Cartilage Oligomeric Matrix Protein (*COMP*) is a non-collagenous protein present in the extracellular matrix of bone and cartilage, and mutation in this gene can cause death of the chondrocytes [52]. Chondrocyte death can be caused by the retention of *COMP* and other extracellular matrix proteins within an enlarged rugged endoplasmic reticulum, and this can lead to an abnormal matrix that is easily eroded over time [53]. In addition, *COMP* may be a potential molecular marker for bone diseases [54–56].

The *CSGALNACT1* gene encodes the N-acetilgalactosaminiltransferase enzyme, which has activity in beginning and the elongation of the chondroitin sulfate synthesis [57]. In rats, reduced levels of *CSGALNACT1* may cause post-natal lethality due to respiratory failure, mild dwarfism and the cartilage has an abnormality of endochondral ossification [58, 59].

Cysteine-Rich Angiogenic Inducer 61 (*CYR61* or *CCN1*) is part of the group of matricellular proteins [60], being associated with extracellular matrix secretion [61]. Furthermore, *CYR61* has important role in regulating inflammation and possible cell repair [62]. This gene potentiates DNA synthesis for cell proliferation induced by other mitogens [63] in response to bacterial or viral infection [64, 65].

Frizzled Related Protein (*FRZB*/*SFRP-3*) is part of the set of Secreted Frizzled-Related Protein (*SFRP1*), which comprises five members that modulate negatively and positively the Wingless-type (Wnt) signaling cascade [66]. Wnt signaling cascade is important for regulating the development, maintenance and homeostasis of bone and cartilage [67]. Therefore, dysregulation of Wnt signaling may lead to the development of osteoarthritis in rodents [67–71].

Hyaluronan and Proteoglycan Link Protein 1 (HAPLN1/CRTL1) is an abundant polysaccharide in the extracellular matrix of cartilage under normal conditions and is involved in cell differentiation and morphogenesis [72–74]. Recent studies have shown that in bone diseases in humans, such as osteoarthritis and allograft transplantation, there is always a low *HAPLN1* expression [75, 76].

The Indian hedgehog (*IHH*) gene is expressed in cartilage, and it is recognized as regulator of bone development and morphogenesis [77]. *IHH* induces the differentiation of the osteogenic cell of the periosteum, being essential in the differentiation of the osteoblasts and in the maintenance of the growth plate, articular cartilage and adjacent endochondral bone formation [78–80]. Jin et al. [81] demonstrated that a deletion in the *IHH* gene was responsible for the Creeper phenotype in broilers, which is characterized by short and stunted legs. In addition, a mutation in this gene causes brachydactyly in humans [82].

Chondromodulin (*CNMD*) regulates the rapid growth of cartilage and vascular invasion prior to the process of cartilage replacement by the endochondral bone [83]. New evidence on the mechanism of differentiation of mesenchymal stem cells (CTMs) into chondrocytes induced by *ChM-I* shows that the main pathways involved in the process are focal adhesion, glycolysis, regulation of the actin cytoskeleton and the ribosome [84]. In osteoarthritis condition, the expression of *ChM-I* is decreased. Therefore, the regulation of this gene in cartilage may be a potential treatment, because it protects the chondrocytes from hypertrophy and delays the progression of osteoarthritis [85].

Retinol-binding protein (*RBP4A*) has the function of transporting vitamin A in the blood, from the liver to the peripheral tissues [86, 87]. Vitamin A plays an important role in the development of various organs, including bone growth [88], contributing to bone health [89, 90], and abnormal levels of Vitamin A may have a negative impact on bone growth [91–94]. Moreover, *RBP4* is expressed during limb growth and this gene is also involved in chondrogenesis, collagen X transcription and bone mineral density [95–97].

Thrombospondin (THBS1) is a glycoprotein that regulates the structure of the extracellular matrix [98] being involved in the protection of chondrocytes, since it exerts pro-chondrogenic and anti-inflammatory function [99]. Furthermore, THBS1 seems to be important in homeostasis and maintenance of bone matrix integrity, and in the regulation of osteoclast formation [100].

From the 26 DE genes enriching the bone and cartilage development BP, 10 (*ALDH1A2, CDH7, GAL3ST1, KIF26B, PLPPR1, PDE6B, SERINC5, TENM2*, *UCHL1* and *WFIKKN2*) appear to have no direct relationship to bone or cartilage formation. However, due to the complexity of these processes, the function of these genes in the regulation of bone cells could still be unknown, especially in chicken.

### Transcription factors

Nine TF associated to downregulated DE genes were found (Fig. 2), being three of those highly connected (*NFATC2, MAFB and HIF1A:TNA*). Several studies have shown that these TF were related to osteoblast and osteoclast differentiation, and osteolysis [101–108] The *NFATC2* plays important role in the immune response, and in the differentiation and regulation of osteoclast growth [109]. Zanoti and Canalis [102] concluded that the activation of *NFATC2* may inhibit the function of osteoblasts and decrease the volume of spongy bone. Furthermore, *NFATC2* was associated with 24 out of 26 DE genes detected as downregulated in the affected chickens (Fig. 2). Moreover, the *MAFB* TF, although expressed selectively in monocytes [110], can be found in several tissues, and has been related to osteolysis in humans and rats [111] by the negative regulation of important cytokines for osteoclast differentiation [105]. *HIF1A* regulates oxygen homeostasis, glucose transport and generation of anaerobic energy in joints and chondrocytes, and may play an important role in the osteoarthritis metabolism [107, 112]. Four other TF were also found with a high number of connections and appear to be associated with osteosarcoma (*EWSR1-FLI1* and *NFIC*), cranium formation (*TCF3*), soft tissue calcification and chondrocyte differentiation (*NF-KAPPAB*) [113–117].

Clinical implications related to BCO include, but are not limited to claudication. There is no efficient treatment and the diagnosis is not possible in early stages, being necessary to perform a necropsy. The DE genes and transcription factors found in this study play a fundamental role in bone and cartilaginous development in broilers. Their low expression in the affected chickens seems to be related to incomplete bone formation, initiating the BCO process in the tibia growth plate of fast-growing chickens. The current findings confirm our hypothesis and indicate that improvement in ossification and cartilage formation have to be addressed in poultry breeding programs.

## Conclusions

We found 16 differentially expressed genes in the tibia GP transcriptome that are directly responsible for bone and cartilage formation, which were downregulated in BCO-affected broilers. According to our data, the lack of ossification might be the main cause of BCO in broilers, and the bacterial process seems to be a secondary condition. Moreover, our results highlight the pathogenesis of BCO, and show that to pursue prevention and control of such condition, breeding strategies have to focus on the improvement of ossification and cartilage formation.

## Methods

### Animals and sample collection

This study was performed at the Embrapa Swine and Poultry National Research Center. Approximately 50 male chicks from Cobb500 commercial broiler line were raised from 1 to 42 days of age, with a density of 12 broilers/m^2^ with infrared lamps. The drinkers, feeders, curtains, light, and chickens were managed following the recommendations for the commercial line. Diets containing 3150 kcal/kg AME and 21% CP (1–21 days), 3200 kcal/kg AME and 20% CP (22–35 days), and 3200 kcal/kg AME and 18.5% CP (36–42 days) were provided. The animals had free access to feed and water. Before sample collection, the broilers were weighted at 42 days of age and euthanized by cervical dislocation followed by bleeding, according to the approval of the Embrapa Swine and Poultry Ethical Committee of Animal Use (CEUA), under protocol number 012/2012.

A classification of the tibia was performed according to the presence or absence of different levels of BCO, by visual observation of compatible necrosis lesions, according to Wideman et al. (2012) [3]. Tibia samples with adhesion between the growth plate (GP) and cartilage (CA) were considered in the normal group and those presenting separation between GP and CA were classified as the affected group. Only those with the initial level of BCO and present in both tibias were used in this study. For sample collection of the normal group, all CA was removed to access the GP. The entire GP of samples were collected, stored in liquid nitrogen and transferred to the − 80 °C freezer for further RNA analysis. Six samples (three normal and three affected) were collected and prepared for RNA-Seq analysis.

### RNA extraction and library preparation

About 100 mg of the tibia GP tissue was used for RNA extraction using Trizol Reagent (Invitrogen, Carlsbad, CA) following the manufacturer’s instructions. The RNA cleanup was performed using Rneasy mini kit (Qiagen, Germany) following manufacturer’s instructions. After RNA extraction, the RNA was quantified in Nanodrop spectrophotometer (Thermo Scientific; Waltham, MA, USA) and the Agilent 2100 BioAnalyzer (Agilent Technologies; Santa Clara, CA, USA) was used for integrity measurement, where samples with RNA integrity number (RIN) higher than 8 were used for library preparation. A total of six tibia samples, three normal and three BCO-affected were prepared for RNA-sequencing using the TruSeq™ RNA Sample Prep Kit v2 (Illumina, Inc.; San Diego CA, USA), according to the manufacturer’s recommendations, with 2 μg of total RNA.

### Sequencing, quality control, assembly and differential expression analysis

The libraries were sent to the Functional Genomics Center, ESALQ, University of São Paulo, Piracicaba, SP, Brazil for sequencing in Illumina HiSeq2500 equipment (Illumina, Inc.; San Diego CA, EUA), all in the same lane, following the 2x100bp paired-end protocol.

The quality control was performed using SeqyClean tool (https://github.com/ibest/seqyclean) with the raw FASTQ data for removing short reads (<70pb), low quality reads (Qphred < 24), PCR artifacts and adapter sequences. The sequence reads were mapped against the chicken reference genome (*Gallus gallus*, assembly 5.0) available in (www.ensembl.org), using BWA-MEM software [118]. The read counting was performed with Htseq software [119] using Ensembl annotation release 89. The edgeR package [120] in R environment [121] was used to identify differentially expressed (DE) genes from BCO-affected and unaffected groups. Significance threshold for DE genes was set at a False Discovery Rate (FDR) ≤ 0.05 after multiple correction tests to reduce type I error. Smear plots of DE genes were generated using the expression data for each gene within each sample in the edgeR package from the R environment [121].

### Gene ontology (GO) and network analyses

In a first step, a total of 192 DE genes with log Fold Change (logFC) < − 2.0 and > 2.0 was used as input on DAVID Bioinformatics Resources 6.8 tool (https://david.ncifcrf.gov/summary), and all the identified expressed genes were used as background. Genes with positive or negative logFC values were considered, respectively, upregulated or downregulated when the BCO-affected group was compared to the unaffected group. In a second step, a GO analysis was performed using only upregulated DE genes (63 genes) and only downregulated DE genes (129 genes).

The gene and transcription factor (TF) network analysis was built using Cytoscape 3.6.1 [16]. The output of GO analysis was used as input on Cytoscape with each gene associated with the correspondent biological process. Twenty-six genes were submitted to the TFM-Explorer program (http://bioinfo.lifl.fr/tfm-explorer/form.php) to identify the TF related to them. From sequence of a set of gene promoters, TFM-Explorer searches for locally overrepresented transcription factor binding sites (TFBS) using weight matrices from JASPAR database [122] to detect all potential TFBS, and extracts significant clusters by calculating a score function.

## Supplementary information

**Additional file 1.** Detailed expressed values of all Differentially Expressed Genes, showing the name and description of each annotated gene, LogFoldChange, logCPM, FDR and gene Ensembl ID.

**Additional file 2.** Detailed table showing genes among all Biological Process.

## Data Availability

All data generated or analyzed during this study are included in this published article (and its additional files). The transcriptome sequences are available in the SRA database with BioProject number PRJNA352716 and biosample numbers: SAMN05992341, SAMN05992340, SAMN05992339, SAMN05992338, SAMN05992337 and SAMN05992336.

## Abbreviations

BCO: Bacterial Chondronecrosis with Osteomyelitis
DE: Differentially Expressed
BP: Biological Processes
lncRNA: Long Non-Coding RNAs
snoRNA: Small Nucleolar RNAs
GO: Gene Ontology
FDR: False Discovery Rate
TF: Transcription Factors
